# Investigating the causal relationship of gut microbiota with GERD and BE: a bidirectional mendelian randomization

**DOI:** 10.1186/s12864-024-10377-0

**Published:** 2024-05-14

**Authors:** Yuan Liu, Jiali Yu, Yuxiao Yang, Bingyu Han, Qiao Wang, Shiyu Du

**Affiliations:** 1 Graduate School of Beijing, University of Chinese Medicine, Beijing, China; 2 Department of Gastroenterology, Chinese Academy of Medical Sciences & Peking Union Medical College, China-Japan Friendship Hospital(Institute of Clinical Medical Sciences), Beijing, China; 3 Department of Gastroenterology, Peking University China-Japan Friendship School of Clinical Medicine, Beijing, China; 4 Department of Traditional Chinese Medicine for Pulmonary Diseases, China-Japan Friendship Hospital, Beijing, China; 5 Department of Gastroenterology, China-Japan Friendship Hospital, Beijing, China

**Keywords:** Gastroesophageal reflux disease, Barrett’s esophagus, Gut microbiota, Mendelian randomization, Mediation analysis

## Abstract

**Background:**

Gut microbiota(GM) have been proven associated with lots of gastrointestinal diseases, but its causal relationship with Gastroesophageal reflux disease(GERD) and Barrett’s esophagus(BE) hasn’t been explored. We aimed to uncover the causal relation between GM and GERD/BE and potential mediators by utilizing Mendelian Randomization(MR) analysis.

**Methods:**

Summary statistics of GM(comprising 301 bacteria taxa and 205 metabolism pathways) were extracted from MiBioGen Consortium(*N* = 18,340) and Dutch Microbiome Project(*N* = 7,738), GERD and BE from a multitrait meta-analysis(N_GERD_=602,604, N_BE_=56,429). Bidirectional two-sample MR analysis and linkage disequilibrium score regression(LDSC) were used to explore the genetic correlation between GM and GERD/BE. Mediation MR analysis was performed for the risk factors of GERD/BE, including Body mass index(BMI), weight, type 2 diabetes, major depressive disorder(MDD), smoking initiation, alcohol consumption, and dietary intake(including carbohydrate, sugar, fat, protein intake), to detect the potential mediators between GM and GERD/BE.

**Results:**

11 bacterial taxa and 13 metabolism pathways were found associated with GERD, and 18 taxa and 5 pathways exhibited causal relationship with BE. Mediation MR analysis suggested weight and BMI played a crucial role in these relationships. LDSC identified 1 taxon and 4 metabolism pathways related to GERD, and 1 taxon related to BE. Specie *Faecalibacterium prausnitzii* had a suggestive impact on both GERD(OR = 1.087, 95%CI = 1.01–1.17) and BE(OR = 1.388, 95%CI = 1.03–1.86) and LDSC had determined their correlation. Reverse MR indicated that BE impacted 10 taxa and 4 pathways.

**Conclusions:**

This study established a causal link between gut microbiota and GERD/BE, and identified the probable mediators. It offers new insights into the role of gut microbiota in the development and progression of GERD and BE in the host.

**Supplementary Information:**

The online version contains supplementary material available at 10.1186/s12864-024-10377-0.

## Introduction

Gastroesophageal reflux disease(GERD) is a prevalent disorder within the digestive system, which refers to the retrograde flow of gastric and duodenal contents into the esophagus, causing damage to the esophageal mucosal tissue and resulting in a series of digestive symptoms, such as acid regurgitation, heartburn, vomiting, chest pain, and other extra-esophageal symptoms [1, 2]. Numerous studies indicated an increasing trend in the incidence of GERD, with a global prevalence rate of 13.98% [3]. Proton pump inhibitors(PPIs) are currently the priority pharmacotherapy for GERD. However, research indicated that approximately 40% of patients, despite undergoing standard acid suppression treatment, did not achieve efficient symptom control. Additionally, prolonged PPI medication presented an elevated risk of adverse reactions, including fractures, gastrointestinal infections, and acute interstitial nephritis [4–6]. As a high-prevalent chronic condition, GERD presents substantial economic and health burdens to patients and society [7]. EAC is a fatal illness with a poor prognosis, exhibiting a five-year survival rate of ≤ 20% [8]. In 5–12% of cases, GERD leads to the repeated proliferation of esophageal cells to form Barrett’s esophagus(BE), which was the only known precancerous lesion for esophageal adenocarcinoma(EAC) [8, 9], and the risk of malignant transformation for BE with high-grade dysplasia can be as high as 7% [10]. Hence, by studying GERD and BE along with their influencing factors, sufficient attention and improvement measures can be implemented, contributing to primary cancer prevention [11].

Gut microbiota(GM) comprises numerous bacteria residing in the human intestinal tract. At the phylum level, GM in healthy individuals is predominantly composed of *Bacteroidetes*, *Firmicutes*, *Proteobacteria*, and *Actinobacteri* [12]. Malfunctions of GM can initiate a spectrum of illnesses, including metabolic disorders, cardiovascular diseases, immune diseases, mental disorders, and various types of cancers [13]. In recent years, research has suggested that GM played an important role in the occurrence and progression of GERD and BE [14, 15]. For instance, Zou et al [14] indicated that Gram-positive(G+) bacteria were prevalent in the normal esophagus, with *Firmicutes* and *Streptococcus* as the most common. On the contrary, Gram-negative(G-) bacteria took precedence in individuals with GERD/BE, and the abundance of *Streptococcus* decreased. The lipopolysaccharides(LPS) presented in G- bacteria can activate Toll-like receptors and NF-kB pathway subsequently promoting the secretion of inflammatory cytokines such as IL-8 and IL-1b.

Simultaneously, epidemiological studies have revealed several potential risk factors for GERD, including obesity [16], smoking [16], alcohol [17], diabetes [18], depression [4] and so on. However, much of the evidence lacked reliability, displaying inconclusive outcomes across various investigations, and the precise cause-and-effect relationship of mediators in these connections was not adequately established. Moreover, in observational research, the presence of confounders, reverse causation, and other mistakes might hinder the establishment of causal inferences [19].

Mendelian randomization(MR) is a method that employs genetic variants as instrumental variables(IVs) to estimate causal relationships between exposure and outcomes. Because genetic loci are determined at conception and remain unaffected by environmental, economic, or cultural factors, MR could help mitigate the impact of confounders [19]. Recently, MR has been widely applied to assess the potential causal relationships between GM and various digestive disorders [20–22].

This research utilized genome-wide association study(GWAS) data and performed a bidirectional Mendelian randomization approach to analyze the causal relationships between GM and GERD/BE. Additionally, we explored whether risk factors mediated the impact of GM on GERD/BE. This research aims to enhance causal inferences in the field of GERD and BE epidemiology, improve the understanding of potential risk factors, and offer valuable insights for future research design and data analysis.

## Methods

### Study design

**Fig. 1:**
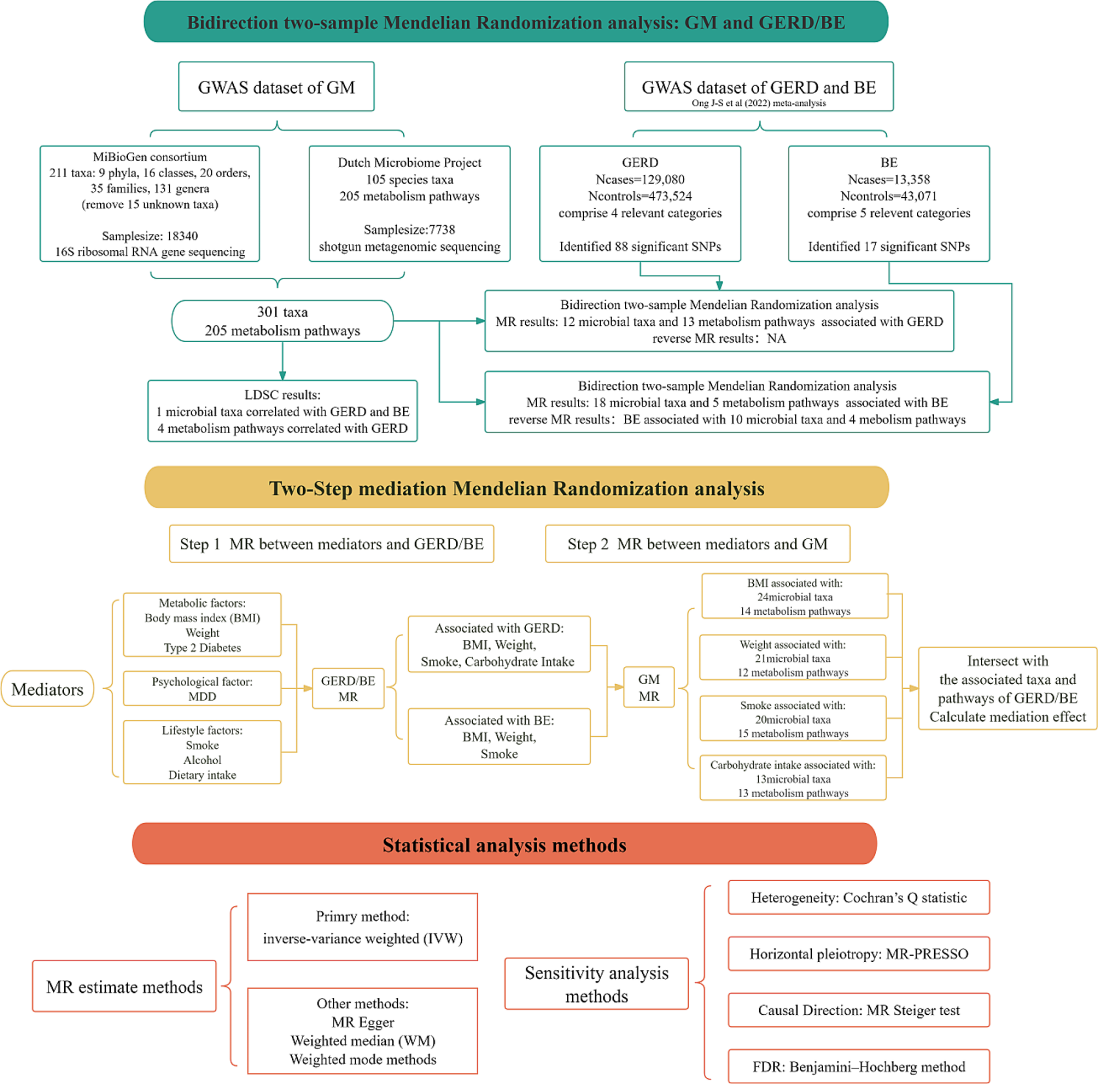
Flowchart of the MR analysis in this study. BE, Barrett’s esophagus; GM, Gut microbiota; GERD, Gastroesophageal reflux disease; LDSC, linkage disequilibrium score regression; MR, Mendelian Randomization; MR-PRESSO, Mendelian Randomization Pleiotropy Residual Sum and Outlier

This bidirectional two-sample MR analysis was designed to explore the potential causality between GM and the risk of GERD/BE, as illustrated in Fig. 1. This research was performed according to the Strengthening the Reporting of Observational Studies in Epidemiology Using Mendelian Randomization(STROBE-MR) checklist [23].

### GWAS data for gut microbiota

Summary statistics for human gut microbiota were obtained from two GWAS datasets (Supplementary Table S1). The statistics from the MiBioGen consortium were curated from 18,340 multiple-ancestries participants via 16 S ribosomal RNA gene sequencing, containing 211 taxa: 9 phyla, 16 classes, 20 orders, 35 families, and 131 genera [24]. After removing 15 unknown families of genera, we included 196 taxa for MR analysis. The statistics on the 105 species level of gut microbiota abundance and 205 gut bacterial pathways abundance were retrieved from the Dutch Microbiome Project(DMP), which was curated from 7738 European-ancestry individuals via shotgun metagenomic sequencing [25]. In general, our MR analysis was based on 301 bacterial taxa and 205 metabolism pathways(Supplementary Table S2-3).

### GWAS data for GERD and BE

The IVs for GERD and BE were acquired from a recently published meta-analysis of GWASs(Supplementary Table S1,4), which is based on European-ancestry and utilized a multitrait analysis framework to expand the genetic loci for GERD and BE [26]. The GERD summary data comprised 129,080 cases and 473,524 controls from 4 relevant categories[UK Biobank(UKB) and QSkin studies for GERD(Ncases = 78,707, Ncontrols = 288,734), meta-analysis combining UKB and GIANT consortium for BMI(*N* = 681,275), Psychiatric Genomics Consortium for MDD(Ncases = 170,756, Ncontrols = 329,443), Social Science Genetic Association Consortium for education attainment(*N* = 766,345)], in which had identified 88 SNPs significantly associated with GERD. The phenotypic definition of GERD included self-report, ICD-10 diagnosis, ICD-9 diagnosis, operative procedures, self-reported GERD symptoms such as heartburn, and the use of GERD-related medication. Similarly, the BE summary data comprised 13,358 cases and 43,071 controls from 5 associated categories[meta-analysis combining UKB, Barrett’s and Esophageal Adenocarcinoma Consortium(BEACON), Bonn, Cambridge and Oxford studies for BE(Ncases = 9,680, Ncontrols = 31,211), besides the other 4 categories had mentioned before for GERD, BMI, MDD, and education attainment], with 17 SNPs identified significant association. BE’s phenotypic definition contains ICD-10 diagnosis and pathologically verified.

### GWAS data for the mediator

In regards to mediation MR analysis, we selected three categories of risk factors that have been proven strongly associated with GERD/BE to represent mediators. Body mass index(BMI), weight, and type 2 diabetes were used for metabolic factors, major depressive disorder(MDD) for psychological factor, smoking initiation(defined as a binary phenotype representing whether participants had ever smoked regularly), alcohol(defined as a continuous phenotype representing alcoholic drinks per week), and dietary intake(including carbohydrate, sugar, fat, protein intake) for lifestyle factors. Summary statistics of these mediators were obtained from respective GWASs [27–31](Supplementary Table S1).

### Instrumental variables selection

The single nucleotide polymorphisms(SNPs) that surpassed the genome-wide significance threshold of *P* < 5 × 10^− 8^ were extracted as IVs for GERD, BE, and mediators. As for GM taxa and pathways, to get more comprehensive results and maximize the number of instruments, SNPs with a threshold *P* < 1 × 10^− 5^ were included. Then, all of the SNPs were clumped to a linkage disequilibrium threshold of r^2^ < 0.001 within a distance of 10,000 kilobases(kb) utilizing the 1000 Genomes European reference panel. The F-statistics for all SNPs were computed using the following formulas: F(primary formula) =[R^2^/K ×(N-K-1)/(1-R^2^)], F(alternative formula, if the database lacks samplesize [32]) =β^2^/SE^2^, and the SNPs with F < 10 were removed. Subsequently, eliminate the taxon or pathway that has fewer than three SNPs [33].

### Statistical analysis

#### Bidirectional Mendelian Randomization between gut microbiota and GERD/BE

The primary aspect of our study was the bidirectional two-sample MR between GM and GERD/BE. To estimate the causal effect of exposure or mediator on outcome, the IVs involved in the two-sample MR need to follow three main assumptions: (1) Instrumental variables are correlated with the exposure. (2) Instrumental variables are unrelated to confounders of the exposure-outcome relationship. (3) Instrumental variables only influence the outcome through exposure and mediators [19]. We used four MR methods to determine the MR estimates between the exposure and the outcome, including the inverse-variance weighted(IVW), MR Egger, Weighted median(WM), and Weighted mode methods. IVW was selected to be the primary method to gain the highest efficiency, and we combined these methods to ensure the robustness of our results. Furthermore, we conduct bivariate linkage disequilibrium score regression(LDSC) to explore the genetic correlation between GM and GERD/BE based on the GWAS statistics, to substantiate our findings.

#### Mediation effect of multiple risk factors between gut microbiota and GERD/BE

To explore the potential mechanisms between GM and GERD/BE, we performed a two-step mediation MR for the previously mentioned risk factors of GERD/BE. Firstly, we conduct a pair-wise two-sample MR to detect the causal relationship between risk factors and GERD/BE. Secondly, we identified the associated taxa and pathways of the mediators by pair-wise MR between GM and mediators, then intersected them with the associated taxa and pathways of GERD/BE, and calculated the mediating effect. We used the coefficients method to estimate the indirect effect of GM on GERD/BE, then divided the indirect effect by the total effect to calculate the mediation effect(β1 × β2/ β3), among which β1 represented the effect of GM on the risk factors, β2 represented the effect of risk factors on GERD/BE, and β3 represented the effect of GM on GERD/BE [34]. In reverse MR analysis, we performed mediation analysis similarly as well.

### Sensitivity analysis

We performed a series of sensitivity analyses to confirm the robustness of our results. Heterogeneity was estimated by Cochran’s Q statistic, and the results with the *P-value* < 0.05 were removed due to their heterogeneity. Furthermore, we conduct The Mendelian Randomization Pleiotropy Residual Sum and Outlier(MR-PRESSO) tests to assess horizontal pleiotropy [35], and the MR Steiger test to ensure the right causal direction from exposure to outcome [36]. The Benjamini‒Hochberg method was used to evaluate the false discovery rate(FDR) and provided corrected FDR *Q-value* [37], which methods have been widely performed in this field. Once the results’ *Q-value* was less than 0.05, it represents a significant association, on the other hand, if the *P-value* is less than 0.05 but the *Q-value* is greater than 0.05, it can be considered as a suggestive association [11]. All of the statistical analyses were accomplished based on R(version 4.3.0) and the R packages of “TwoSampleMR” and “Mendelian Randomization”, GraphPad Prism (version 8.0.1.244) were used to analyze and export relevant volcano plots and forest plots.

## Results

### Instrumental variables

In our research, there are 1184 SNPs for MiBioGen gut microbiota and 1575 SNPs for DMP gut microbiota. Specifically, the number of SNPs for each gut microbiota taxon abundance or gut bacterial pathway abundance ranges from 3 to 33. Meanwhile, there are 88 SNPs and 17 SNPs for GERD and BE, respectively. The details of SNPs of GM, mediators, GERD, and BE were summarized in Supplementary Tables S2-4. The F-statistics for all SNPs involved in our research ranged from 18.69 to 321.31, greater than 10, which indicated the robustness of our instrumental variables.

### MR analysis of GM on GERD

#### MR analysis of gut microbiota abundance on GERD

**Fig. 2:**
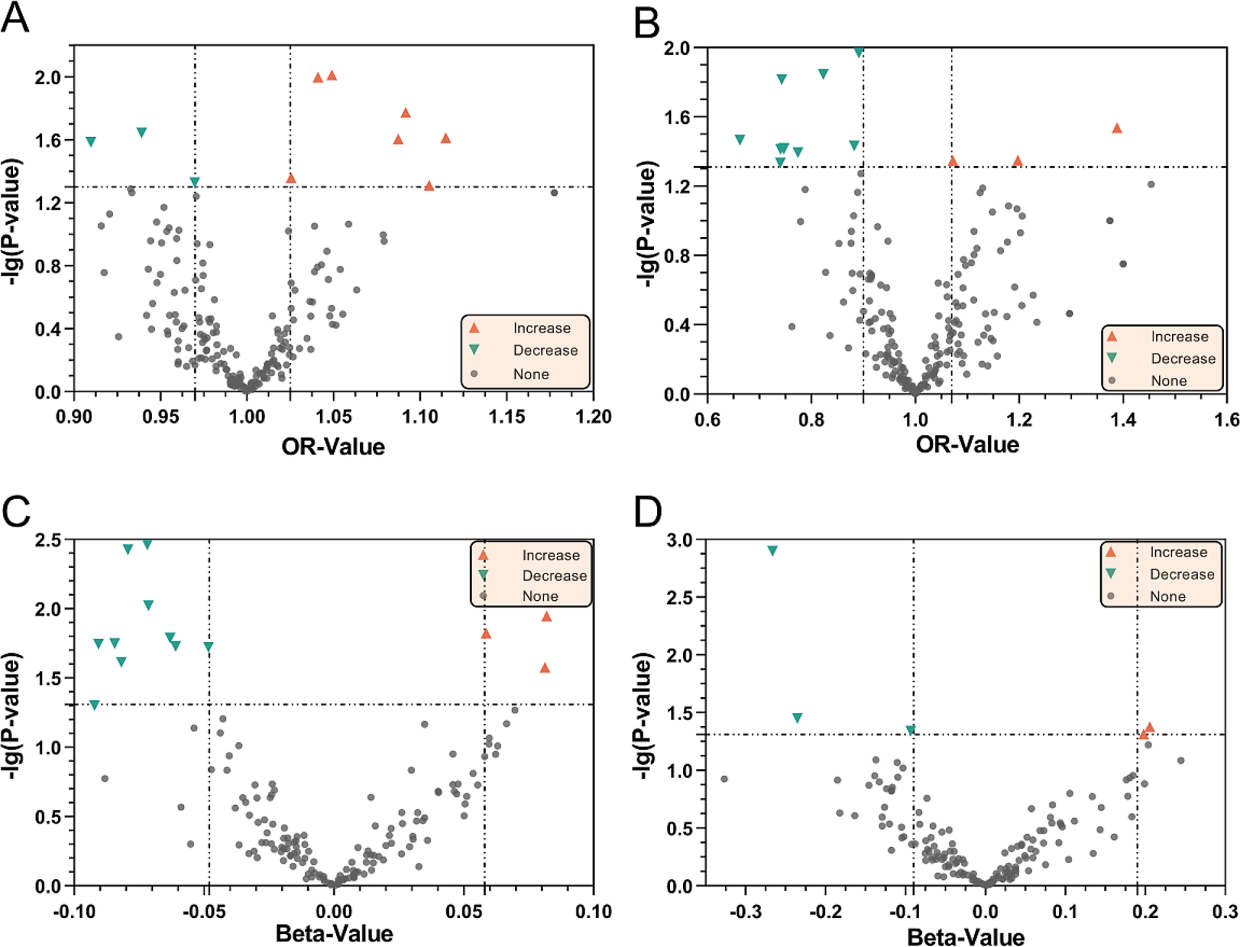
The volcano plot of the MR analysis Inverse-variance weighted method results between GM and GERD/BE. The X-axis represents the OR/beta-value, the Y-axis represents the logarithmic p-value with a base of 10. The red triangles represent the GM taxa or metabolism pathways increase the risk of GERD/BE, while green triangles represent the opposite meaning, the gray circle represent no effect taxa/pathways. **A** MR analysis results indicated the causal relationship between GM taxa and GERD. **B** MR analysis of GM taxa on BE. **C** MR analysis of GM metabolism pathways on GERD. **D** MR analysis of GM metabolism pathways on BE

**Fig. 3:**
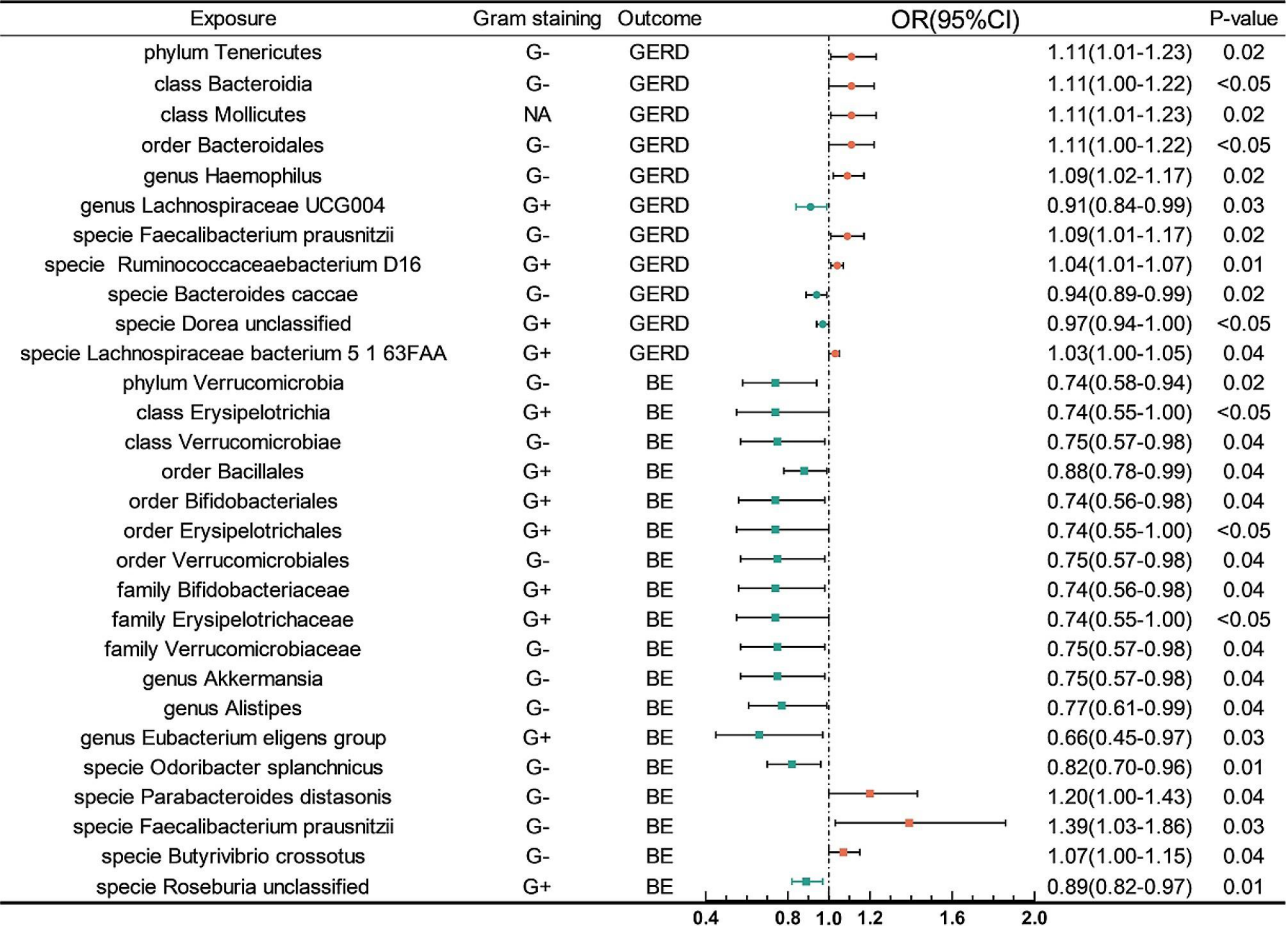
Forest plot of the MR analysis(Inverse-variance weighted method) between GM taxa and GERD/BE. CI, confidence interval; OR odds ratio

We identified 18 microbial taxa suggestively associated with GERD, 6 taxa were excluded because the SNPs were less than 3(Figs. 2 and 3, Supplementary Table S5), and 1 taxa was excluded because of horizontal pleiotropy. The IVW analysis indicated that phylum *Tenericutes*(OR: 1.11, 95% CI: 1.01–1.23, *P* = 0.02), class *Bacteroidia*(OR: 1.10, 95% CI: 1.00-1.22, *P* < 0.05), class *Mollicutes*(OR: 1.11, 95% CI: 1.01–1.23, *P* = 0.02), order *Bacteroidales*(OR: 1.11, 95% CI: 1.00-1.22, *P* < 0.05), genus *Haemophilus*(OR: 1.09, 95% CI: 1.02–1.17, *P* = 0.02), specie *Eubacterium hallii*(OR: 1.05, 95% CI: 1.01–1.09, *P* = 0.01), *specie Faecalibacterium prausnitzii*(OR: 1.09, 95% CI: 1.01–1.17, *P* = 0.02), specie *Ruminococcaceae bacterium D16*(OR: 1.04, 95% CI: 1.01–1.07, *P* = 0.01), and specie *Lachnospiraceae bacterium 5 1 63FAA*(OR: 1.03, 95% CI: 1.00-1.05, *P* = 0.04) were suggestive associated with an increasing risk of GERD, meanwhile, genus *Lachnospiraceae UCG004*(OR: 0.91, 95% CI: 0.84–0.99, *P* = 0.03), specie *Bacteroides caccae*(OR: 0.94, 95% CI: 0.89–0.99, *P* = 0.02), and specie *Dorea unclassified*(OR: 0.97, 95% CI: 0.94-1.00, *P* < 0.05) were suggestive associated with a decreasing risk of GERD. However, only *Mollicutes*, *Haemophilus*, *Tenericutes*, *Ruminococcaceae bacterium D16*, *Lachnospiraceae UCG004*, *Bacteroides caccae*, *Dorea unclassified*, and *Lachnospiraceae bacterium 5 1 63FAA* maintained the same direction results in MR Egger, WM, and Weighted mode methods. Sensitivity analysis(Supplementary Table S13) illustrated that the direction of these MR estimates was supported by the MR Steiger test, and heterogeneity was not found in all of these results. Horizontal pleiotropy was only detected on *Eubacterium hallii*(p-value of Egger intercept: 0.048).

#### MR analysis of gut microbiota metabolism pathways on GERD

**Fig. 4:**
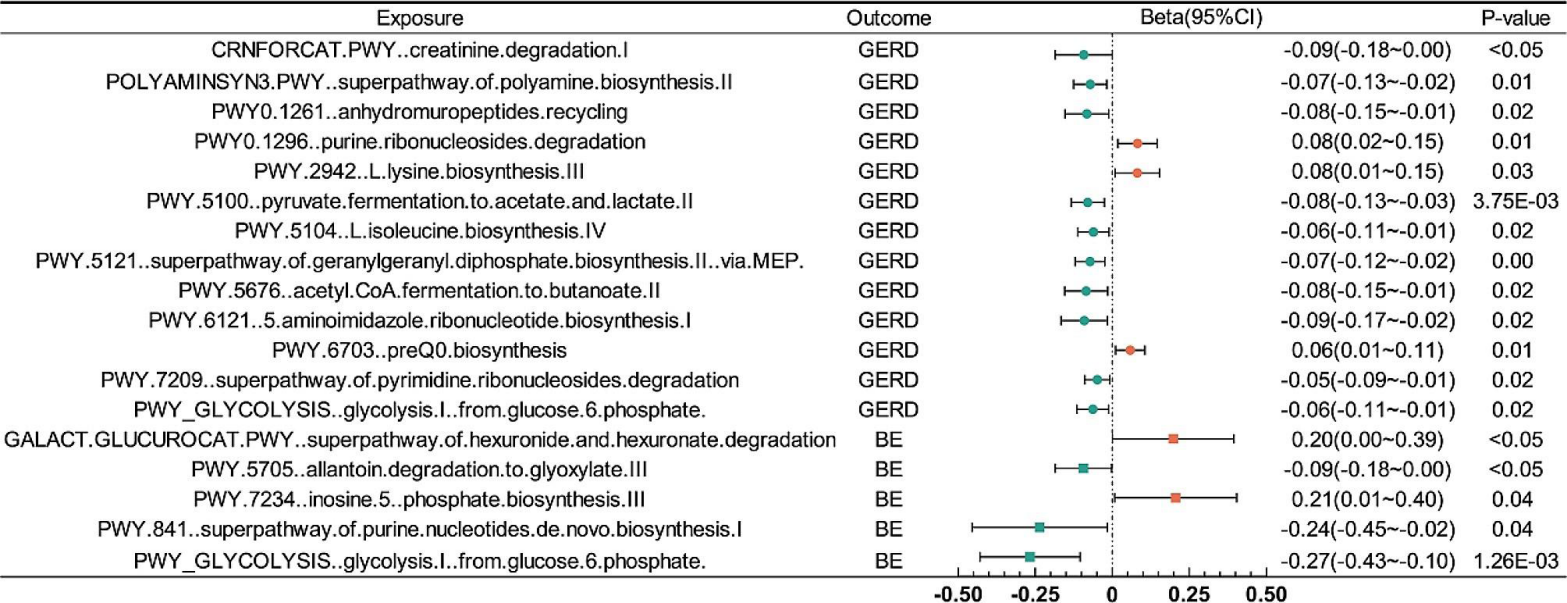
Forest plot of the MR analysis(Inverse-variance weighted method) between GM metabolism pathways and GERD/BE. CI, confidence interval

We identified that a per unit increased abundance of 13 metabolism pathways had suggestive association on GERD(Figs. 2 and 4, Supplementary Table S5), among which, *purine ribonucleosides degradation*(b: 0.08, 95% CI: 0.02 ∼ 0.15, *P* = 0.01) associated with the highest increasing risk of GERD, and *creatinine degradation I*(b:-0.09, 95% CI:-0.18∼-2.01E-05, *P* < 0.05) associated with the highest decreasing risk of GERD.

### MR analysis of GM on BE

#### MR analysis of gut microbiota abundance on BE

23 microbial taxa were defined as suggestively associated with BE(Figs. 2 and 3, Supplementary Table S5), and 5 taxa were excluded because of the lack of enough SNPs. The IVW analysis indicated that specie *Parabacteroides distasonis*(OR: 1.20, 95% CI: 1.00-1.43, *P* = 0.04), specie *Faecalibacterium prausnitzii*(OR: 1.39, 95% CI: 1.03–1.86, *P* = 0.03), and specie *Butyrivibrio crossotus*(OR: 1.07, 95% CI: 1.00-1.15, *P* = 0.04) were suggestive associated with an increasing risk of GERD. Similarly, phylum *Verrucomicrobia*(OR: 0.74, 95% CI: 0.58–0.94, *P* = 0.02), class *Erysipelotrichia*(OR: 0.74, 95% CI: 0.55-1.00, *P* < 0.05), class *Verrucomicrobiae*(OR: 0.75, 95% CI: 0.57–0.98, *P* = 0.04), order *Bacillales*(OR: 0.88, 95% CI: 0.78–0.99, *P* = 0.04), order *Bifidobacteriales*(OR: 0.74, 95% CI: 0.56–0.98, *P* = 0.04), order *Erysipelotrichales*(OR: 0.74, 95% CI: 0.55-1.00, *P* < 0.05), order *Verrucomicrobiales*(OR: 0.75, 95% CI: 0.57–0.98, *P* = 0.04), family *Bifidobacteriaceae*(OR: 0.74, 95% CI: 0.56–0.98, *P* = 0.04), family *Erysipelotrichaceae*(OR: 0.74, 95% CI: 0.55-1.00, *P* < 0.05), family *Verrucomicrobiaceae*(OR: 0.75, 95% CI: 0.57–0.98, *P* = 0.04), genus *Akkermansia*(OR: 0.75, 95% CI: 0.57–0.98, *P* = 0.04), genus *Alistipes*(OR: 0.77, 95% CI: 0.61–0.99, *P* = 0.04), genus *Eubacterium eligens group*(OR: 0.66, 95% CI: 0.45–0.97, *P* = 0.03), specie *Odoribacter splanchnicus*(OR: 0.82, 95% CI: 0.70–0.96, *P* = 0.01), and specie *Roseburia unclassified*(OR: 0.89, 95% CI: 0.82–0.97, *P* = 0.01) were suggestive associated with a decreasing risk of BE. Whereas, sensitivity analysis(Supplementary Table S13) demonstrated that only *Erysipelotrichia*, *Bifidobacteriaceae*, *Erysipelotrichaceae*, *Alistipes*, *Bifidobacteriales*, *Erysipelotrichales*, *Odoribacter splanchnicus*, *Faecalibacterium prausnitzii*, and *Roseburia unclassified* remained the same direction in other three methods. The Steiger test substantiated these MR estimates, demonstrating the absence of heterogeneity and horizontal pleiotropy.

#### MR analysis of gut microbiota metabolism pathways on BE

5 metabolism pathways were identified had suggestive association on BE(Figs. 2 and 4, Supplementary Table S5). *Phosphate biosynthesis III*(b: 0.21, 95% CI: 0.01 ∼ 0.40, *P* = 0.04) associated with the highest increasing risk of BE, and *glucose 6 phosphate*(b:-0.27, 95% CI:-0.43∼-0.10, *P* = 1.26E-03) associated with the highest decreasing risk of BE.

### LDSC results between GM and GERD/BE

LDSC results(Supplementary Table S6) supported the genetic correlation between 4 pathways and GERD, meanwhile, 1 GM taxa(specie *Faecalibacterium prausnitzii*) correlated with both GERD(RG: -0.44, *P* = 3.45E-06) and BE(RG: 0.15, *P* = 0.34).

### Reverse MR analysis of GERD/BE on GM

#### MR analysis of GERD on GM

We performed reverse MR analysis of GERD on GM(Supplementary Table 7), which indicated that GERD had no causal effect on microbiota taxa or metabolism pathways.

#### MR analysis of BE on GM

**Fig. 5:**
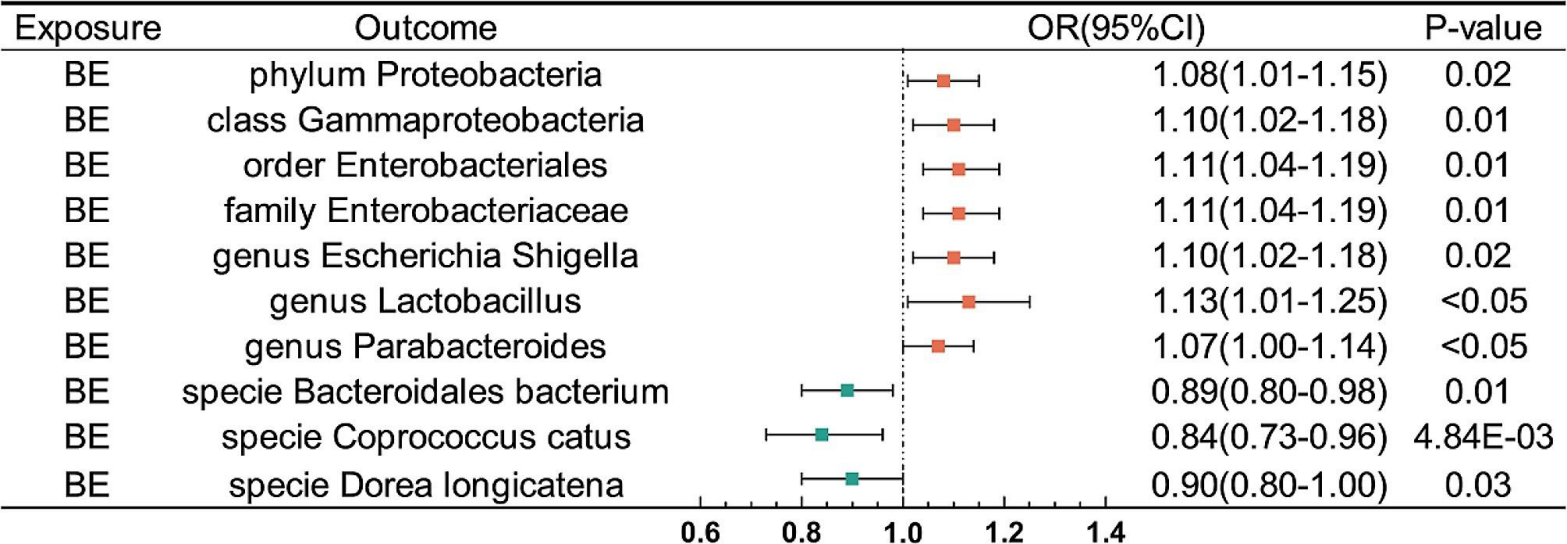
Forest plot of the reverse MR analysis(Inverse-variance weighted method) between BE and GM taxa. CI, confidence interval; OR odds ratio

BE was defined as suggestively associated with 10 taxa(Fig. 5; Tables 1 and 2). The IVW analysis indicated that BE were suggestively associated with an increasing risk of phylum *Proteobacteria*(OR: 1.08, 95% CI: 1.01–1.15, *P* = 0.02), class *Gammaproteobacteria*(OR: 1.10, 95% CI: 1.02–1.19, *P* = 0.01), order *Enterobacteriales*(OR: 1.11, 95% CI: 1.03–1.20, *P* = 0.01), family *Enterobacteriaceae*(OR: 1.11, 95% CI: 1.03–1.20, *P* = 0.01), genus *Escherichia Shigella*(OR: 1.10, 95% CI: 1.01–1.19, *P* = 0.02), genus *Lactobacillus*(OR: 1.13, 95% CI: 1.00-1.28, *P* < 0.05), and genus *Parabacteroides*(OR: 1.07, 95% CI: 1.00-1.15, *P* < 0.05). Whereas, BE was suggestively associated with a decreasing risk of specie *Bacteroidales bacterium ph8*(OR: 0.89, 95% CI: 0.81–0.98, *P* = 0.01), specie *Coprococcus catus*(OR: 0.84, 95% CI: 0.75–0.95, *P* = 4.84E-03), and specie *Dorea longicatena*(OR: 0.90, 95% CI: 0.81–0.99, *P* = 0.03). As for metabolism pathways, BE was identified as having a suggestive association with 4 pathways. BE had the highest causal effect on increasing risk of *thiamin salvage II*(b: 0.09, 95% CI: 0.01–0.18, *P* = 0.03), and the decreasing risk of *tRNA charging*(b: -0.09, 95% CI: -0.18∼-9.67E-04, *P* < 0.05). These MR estimations were validated using the Steiger test, and no evidence of heterogeneity or horizontal pleiotropy was detected.

### Mediation MR analysis between GM and GERD/BE

We performed a two-step MR analysis to estimate the causal effect of the mediators. Firstly, by conducting pair-wise MR between risk factors and GERD/BE, we identified that four factors, including BMI, weight, carbohydrate intake, and smoking were associated with GERD. Meanwhile, three mediators namely BMI, weight, and smoking were associated with the BE, as shown in Supplementary Tables 10–11. Secondly, the intersection result exhibited that only BMI and weight had the mediation effect in our research(Table 3, Supplementary Table 12). Specifically, genus *Lachnospiraceae UCG004* was associated with weight(OR: 0.97, 95% CI: 0.95-1.00, *P* = 0.04), and the proportion of the mediation effect of genus *Lachnospiraceae UCG004* on GERD via weight was 11.96%(95% CI: 1.69–12.61%, *P* < 0.05). Specie *Odoribacter splanchnicus* and specie *Parabacteroides distasonis* were associated with BMI(OR: 0.98, 95% CI: 0.96–0.99, *P* = 0.01; OR: 1.02, 95% CI: 1.00-1.03, *P* = 0.04), and the proportion of the mediation effect on BE via BMI were 12.09%(95% CI: 11.94–13.40, *P* = 0.01) and 8.94%(95% CI: 6.16–8.97, *P* < 0.05), respectively. As for metabolism pathways, *superpathway of geranylgeranyl diphosphate biosynthesis II via MEP* was proved significantly associated with BMI(b:-0.03, 95% CI:-0.05∼-0.02, *P* = 3.54E-06, *Q* = 1.77E-04) and weight(b:-0.03, 95% CI:-0.04∼-0.01, *P* = 6.29E-05, *Q* = 3.14E-03), and the proportion of the mediation effect on GERD via BMI and weight was 28.62%(95% CI: 25.37–45.10, *P* = 4.41E-05) and 13.45%(95% CI: 12.73–17.12, *P* = 6.98E-04), respectively. As for reverse MR, the mediation effect was not detected.

**Table 1:** Reverse MR analysis results of BE on gut microbiota taxa

Exposure	Outcome	Gram staining	Method	OR	95%CI	*P*-value
BE	phylum Proteobacteria	G-	IVW	1.08	1.01–1.15	0.02
BE	class Gammaproteobacteria	G-	IVW	1.10	1.02–1.19	0.01
BE	order Enterobacteriales	G-	IVW	1.11	1.03–1.20	0.01
BE	family Enterobacteriaceae	G-	IVW	1.11	1.03–1.20	0.01
BE	genus Escherichia Shigella	G-	IVW	1.10	1.01–1.19	0.02
BE	genus Lactobacillus	G+	IVW	1.13	1.00 -1.28	< 0.05
BE	genus Parabacteroides	G-	IVW	1.07	1.00 -1.15	< 0.05
BE	specie Bacteroidales bacterium ph8	G-	IVW	0.89	0.81–0.98	0.01
BE	specie Coprococcus catus	G+	IVW	0.84	0.75–0.95	4.84E-03
BE	specie Dorea longicatena	G+	IVW	0.90	0.81–0.99	0.03

## Discussion

The human body is an integrity system, where the gut microbiota and esophagus are correlative and inseparable. Multiple studies [38–40] have shown strong genetic evidence supporting the reciprocal relationships between GM and GERD/BE. GM has the potential to predict the histological alterations that occur during GERD and BE progresses, such as inflammation, carcinogenesis, hyperplasia, and metaplasia [41]. Esophageal microbiota’s relationship with GERD/BE was characterized by a transition from the Type I to the Type II flora [42]. Although the action ratio and efficacy of G + and G- bacteria in the development of GERD and BE were nearly the same [15, 43], type II flora was rich in G- bacteria and mostly associated with GERD and BE, while type I flora was predominantly composed of G + bacteria and tied to normal esophageal function [44]. This signifies a transition from a condition characterized by a substantial abundance of G + bacteria to a greater abundance of G- bacteria, accompanied with a reduction in microbial diversity [45, 46].

**Table 2:** Reverse MR analysis results of BE on gut microbiota metabolism pathways

Exposure	Outcome	Method	Beta	95%CI	*P*-value
BE	PWY.6121.5.aminoimidazole.ribonucleotide.biosynthesis.I	IVW	-0.09	-0.17 ∼-0.01	0.04
BE	PWY.6897.thiamin.salvage.II	IVW	0.09	0.01 ∼ 0.18	0.03
BE	PWY.7234.inosine.5.phosphate.biosynthesis.III	IVW	0.09	5.35E-04 ∼ 0.18	< 0.05
BE	TRNA.CHARGING.PWY.tRNA.charging	IVW	-0.09	-0.18 ∼-9.67E-04	< 0.05

**Table 3:** Mediation MR analysis results

Exposure	Category	Outcome	Mediator	Median OR	OR 95%CI	Median Effect	Median Effect 95%CI
genus Lachnospiraceae UCG004	GM taxa	GERD	weight	0.989	0.978-1.000	0.120	0.017 ∼ 0.126
specie Odoribacter splanchnicus	GM taxa	BE	BMI	0.977	0.959–0.995	0.121	0.119 ∼ 0.134
specie Parabacteroides distasonis	GM taxa	BE	BMI	1.016	1.000-1.033	0.089	0.062 ∼ 0.090
GALACT.GLUCUROCAT.PWY.superpathway.of.hexuronide.and.hexuronate.degradation	GM pathway	BE	weight	0.989	0.980–0.998	-0.057	-0.171 ∼-0.006
PWY.5121.superpathway.of.geranylgeranyl.diphosphate.biosynthesis.II.via.MEP.	GM pathway	GERD	weight	0.990	0.985–0.996	0.134	0.127 ∼ 0.171
PWY.5121.superpathway.of.geranylgeranyl.diphosphate.biosynthesis.II.via.MEP.	GM pathway	GERD	BMI	0.950	0.904–0.998	0.286	0.254 ∼ 0.451

Our research demonstrated the similar results in gut microbiota taxa. In the results of genera and species levels, the ratio of G+/G- bacteria associated with an increased risk of GERD was 2:2(G+: specie *Ruminococcaceae bacterium D16*, and specie *Lachnospiraceae bacterium 5 1 63FAA*, G-: genus *Haemophilus* and specie *Faecalibacterium prausnitzii*), while associated with a decreased risk of GERD was 2:1(G+: genus *Lachnospiraceae UCG004* and specie *Dorea unclassified*, G-:specie *Bacteroides caccae*). Conversely, as for taxa associated with increasing risk of BE, G- bacteria taxa demonstrated a greater predominance, with a ratio of 0:3(G-: specie *Faecalibacterium prausnitzii*, specie *Parabacteroides distasonis*, and specie *Butyrivibrio crossotus*), while decreasing risk of BE was 2:3(G+:genus *Eubacterium eligens group* and specie *Roseburia unclassified*, G-: genus *Akkermansia*, genus *Alistipes*, and specie *Odoribacter splanchnicus*). Despite a disparity in the G+/G- ratio of pathogenic bacteria associated with GERD compared to previous esophageal research, a discernible trend of GM emerged with the progression of GERD to BE, highlighting the increasing dominance of G- species.

One of the G- bacteria is worth noting, the species *Faecalibacterium prausnitzii* has pathogenic effects on both GERD(OR = 1.087, 95%CI = 1.01–1.17) and BE(OR = 1.388, 95%CI = 1.03–1.86), and LDSC has determined their correlation, suggesting that it could be used as microbial research targets for esophageal precancerous lesions. However, existing studies have considered *F. prausnitzii* as a new-generation probiotic [47, 48] and served as an indicator or biomarker of intestinal health for Crohn’s disease [49]. *F. prausnitzii’s* high production of butyrate exhibited anti-inflammatory properties by reducing pro-inflammatory cytokines and gastrointestinal mucosal permeability [50, 51], thereby preventing bacterial endotoxin lipopolysaccharide(LPS)-mediated inflammation [52]. Meanwhile, research has demonstrated that *F. prausnitzii* engages with the host epithelial cells, and through inducing a tolerogenic cytokine profile attenuates the inflammatory response [53, 54]. Our research is a breakthrough discovery of this microbiota, further investigations are required to elucidate the particular mechanism by which *F. prausnitzii* may contribute to the pathogenicity of GERD/BE.

The phylum *Firmicutes* and *Bacteroidetes* played a vital role in our research, they could catabolize carbohydrates in the colon to produce SCFAs, which exhibited anti-inflammatory properties by reducing pro-inflammatory cytokines and gastrointestinal mucosal permeability, thereby preventing inflammation mediated by the bacterial endotoxin LPS [55–57]. The genus *Eubacterium* can govern the production of bile acids by regulating the expression of several enzymes involved in their metabolism, such as 7α-hydroxylase(Cyp7a1), oxysterol 7α-hydroxylase(Cyp7b1), and sterol 27-hydroxylase(Cyp27a1) [58]. Our results add to the causal evidence that the genus *Eubacterium* may have a protective role in the development of BE. Bacteria like *Lactobacillus* and *Bifidobacterium* were responsible for the immune response affecting pathogens, producing short-chain fatty acids such as lactic acid [59]. Moreover, it has been shown that these bacteria could interact with stomach mucosal receptors, accelerating gastric emptying and relaxing the lower esophageal sphincter relaxation [60, 61]. Going deeper, refluxed acid and bile salts stimulated NADPH oxidase to generate H_2_O_2_, which activates IKKβ that in turn activates the IκB-NF-κB-PKAc complex through phosphorylation of IκB. This led to the degradation of IκB, which released the p50/p65 heterodimer. PKAc in the activated IκB-NF-κB-PKAc complex phosphorylates p65, inducing further formation of p50/p65 heterodimers, which translocated to the nucleus to stimulate transcription of NF-κB p50 target genes(e.g.CDX2) that might play a role in columnar metaplasia [62], one of the pathophysiological mechanisms of GERD. Building upon prior studies, we proposed a hypothesis: GM is involved in the metabolism of short-chain fatty acids and bile acids, which in turn influence the development of GERD and BE, even EAC further. Except for the GM taxa mentioned before, we also found other taxa that had beneficial or detrimental effects on GERD/BE, further studies are required to validate our findings and investigate the underlying mechanism.

In reverse MR analysis, we found that BE had a promoting effect on the genus *Lactobacillus*, which could produce lactate from the fermentation of carbohydrates and further acidify the microenvironment [63]. In a study that utilized the Cytosponge technique together with other tissue samples to assess the microbial profile throughout various phases of Barrett’s carcinogenesis, the elevated presence of *Lactobacillus fermentum* was observed [46].

The observed pattern of familial clustering between GERD and BE suggested that the genetic component of BE may be influenced by the cause of these two diseases. Some of the risk factors have been discussed in the previous studies [64]. For example, a positive correlation was found between the incidence of esophagitis and an elevated BMI(≥ 25 kg/m^2^), high-fat diets have been associated with changes in the microbiota and esophageal dysplasia in animal studies [65]. Our study proved that BMI and weight could mediate the effect of certain taxa and pathways on GERD/BE. However, there is no evidence to support the role of type 2 diabetes, MDD, smoking initiation, alcohol, and dietary intake played the mediating effect between GM and GERD/BE. A previous study indicated that obese individuals have a lower *Firmicutes*/*Bacteroidetes* ratio [66], we found that the species *Parabacteroides distasonis(Bacteroidetes*) and *the* species *Odoribacter splanchnicus(Bacteroidetes*) used BMI as an intermediary factor, whereas genus *Lachnospiraceae UCG004(Firmicutes*) used weight as mediation factor. The overproduction of fatty acids by a commensal species called *Fusimonas intestini*, which belongs to the family *Lachnospiraceae*, might exacerbate obesity. In mice subjected to a high-fat diet and colonized by *Fusimonas intestini*, there was an observed increase in the expression of proinflammatory genes such as TNF-α, LPS-binding protein, and leptin—markers associated with low-grade inflammation [67]. Additionally, research indicated a positive correlation between BMI and the presence of the family *Lachnospiraceae*, including genera *Blautia*, *Dorea*, and *Ruminococcus* [68]. These associations may shed light on the connection between weight and GERD/BE.

The aforementioned findings demonstrated an intense causal connection between GM and GERD/BE, highlighting the need for further investigation into their specific mechanisms. Moreover, considering the potential of personalized treatment strategies, it is worth considering tailoring therapies based on an individual’s distinct microbial composition [69]. These bacteria could act as a hallmark of disease progression, like a signature of altered microbiota, and they have the potential to become a biomarker for diagnosis, similar to the observed improvement in colorectal cancer surveillance by the identification of *F. nucleatum* [70]. Furthermore, understanding the specific microbiota targeted allows us to leverage the Bacterial Whole-Cell Biosensors(BWCB) method for disease detection and diagnosis. Currently, there is a lack of information regarding GERD and BE within the BWCB framework [71].

The major advantage of our study is that it is the first to thoroughly analyze the potential causal relationships between 301 microbial taxa, 205 metabolism pathways, and GERD/BE using the two-sample MR method. Our analysis provided genetic evidence for a potential causal relationship between GM and GERD/BE, and suggested the potential mediators involved. Performing the MR method had the following advantages. Firstly, it follows Mendel’s Laws of Inheritance, ensuring that alleles are randomly distributed among descendants and are not influenced by diseases, similar to randomization in randomized controlled trials(RCT) [19]. Thus, causal inference is unlikely to be influenced by reverse causality and confounders. Secondly, we performed MR analysis based on the most extensive and up-to-date European population-based GWAS study of GERD and BE. Thirdly, we included six hierarchical levels, ranging from phylum to species, in our collection of GM, which could enhance our ability to comprehensively comprehend the effect of GM and facilitate future investigations into underlying mechanisms. Lastly, we conducted a mediation analysis to help comprehend the potential mechanism linking GM and GERD/BE.

However, our study had several limitations. First, since SNPs with *P* < 5 × 10^− 8^ were too limited for the gut microbiota database, we selected SNPs with *P* < 1 × 10^− 5^ as GM IVs. To obtain reliable IVs, we performed a series of IV screening steps, including excluding SNPs with F < 10 to avoid weak IVs bias and linkage disequilibrium test. Second, whereas our study encompassed 301 microbial taxa, the potential causal relationships of numerous other microbial taxa with GERD/BE were not investigated. Third, this manuscript was a correlation analysis of GM and GERD/BE without explaining the mechanism. Fourth, the MR analysis may be affected by potential pleiotropy. Of note, all exposures in our MR analysis had 3 or more IVs, which may mitigate the impact of potential pleiotropy to some extent, because pleiotropy is unlikely to generate the same association for different IVs [19]. Fifth, the participants in the present study were mostly of European ancestries, and the contribution of host genetics in shaping microbiome composition is unclear [72], which may restrict the generalizability of the findings to different populations. Furthermore, although the Mendelian randomization analysis was comparable to the level of evidence from the RCT study, translating current research findings into clinical practice still requires further research to understand the function of GM and how it interacts with other host factors such as genetics, diet, and lifestyle.

## Conclusion

In general, our bidirectional two-sample MR analysis identified 11 gut microbial taxa and 13 pathways associated with GERD, meanwhile 18 taxa and 5 pathways associated with BE. Reverse MR indicated that BE impacted 10 taxa and 4 pathways. BMI and weight were detected as mediators in Mediation MR analysis. Our research provided fresh perspectives on the role of gut microbiota in host GERD and BE pathogenesis and progress. Further studies are required to explore these potential mechanisms and guide treatment strategies for reducing disease burden.

### Electronic supplementary material

Below is the link to the electronic supplementary material.

Supplementary Material 1

## Data Availability

All of the GWAS data involved in our manuscript is publicly available. The main relevant data is included in the manuscript and supplemental file, further inquiries can be directed to the corresponding authors.
